# Lenvatinib promotes hepatocellular carcinoma pyroptosis by regulating GSDME palmitoylation

**DOI:** 10.1080/15384047.2025.2532217

**Published:** 2025-07-13

**Authors:** Yuan Yuan, Mu-Ru Wang, Yang Ding, Ya Lin, Ting-Ting Xu, Xing-Xing He, Pei-Yuan Li

**Affiliations:** a Division of Gastroenterology, Tongji Hospital, Tongji Medical College, Huazhong University of Science and Technology, Wuhan, China; b Department of Gastroenterology, Zhongnan Hospital of Wuhan University, Wuhan, China; c Department of Gastroenterology, Zhengzhou University People’s Hospital, Zhengzhou, Henan, PR China; d Hubei Provincial Clinical Research Center for Intestinal and Colorectal Diseases, Hubei Key Laboratory of Intestinal and Colorectal Diseases, Wuhan, China; e Department of Gastroenterology, Wenchang People’s Hospital, Hainan, China

**Keywords:** Hepatocellular carcinoma, Lenvatinib, pyroptosis, palmitoylation, GSDME

## Abstract

Lenvatinib, as a multi-kinase inhibitor, has been approved as a first-line drug for patients with advanced hepatocellular carcinoma (HCC). Gasdermin E (GSDME)-mediated pyroptosis, a form of programmed cell death, can be induced by chemotherapy drugs or certain kinase inhibitors. However, the role of Lenvatinib in inducing pyroptosis in HCC warrants further investigation. Phase contrast microscopy, LDH assays, and gain- and loss-of-function strategies were used to evaluate Lenvatinib-induced pyroptosis in HCC cells. GSDME palmitoylation was assessed via the acyl-biotin exchange method. In vivo, a subcutaneous HCC xenograft model in nude mice were established to assess the effects of interfering with GSDME on the sensitivity of HCC to Lenvatinib. Lenvatinib induced pyroptosis in HCC cells in a dose- and time-dependent manner. Additionally, Lenvatinib promoted GSDME cleavage, with upregulation of GSDME enhancing pyroptosis and downregulation reducing this effect. The ABE method revealed that GSDME is palmitoylated, and Lenvatinib increased its palmitoylation, promoting plasma membrane localization and enhancing protein stability. Inhibition of GSDME palmitoylation by 2-BP blocked Lenvatinib-induced pyroptosis. In vivo, upregulation of GSDME increased HCC sensitivity to Lenvatinib and inhibited tumor growth. Lenvatinib induces pyroptosis in HCC by promoting the palmitoylation of GSDME, enhancing its localization to the plasma membrane and increasing its protein stability. Interfering with GSDME, both in vitro and in vivo, affects Lenvatinib-induced pyroptosis, thereby altering the therapeutic sensitivity of HCC to Lenvatinib. Targeting GSDME palmitoylation represents a potential therapeutic strategy for HCC, as it enhances Lenvatinib-induced pyroptosis and improves the therapeutic response.

## GRAPHICAL ABSTRACT

**Figure:**
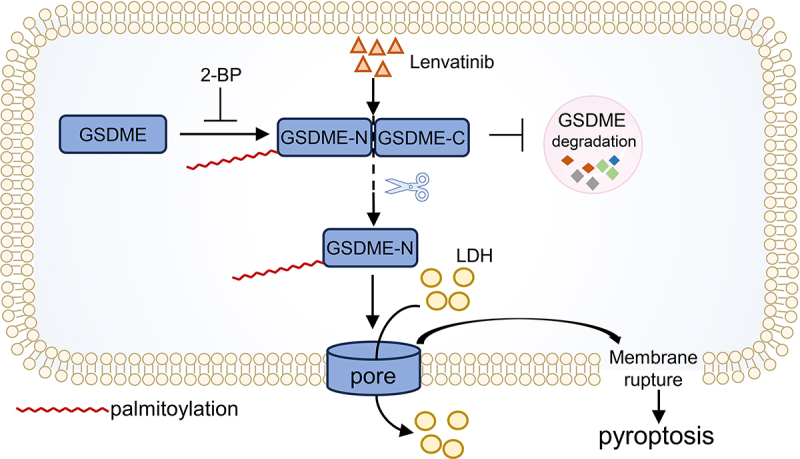

## Introduction

Hepatocellular carcinoma (HCC) is one of the most prevalent and deadly human malignancies worldwide, characterized by strong invasiveness, high malignancy, and a high recurrence rate, posing a significant threat to human health.^1^ Due to the insidious onset, lack of effective early diagnostic methods, and limited treatment options for advanced HCC patients, the overall prognosis for patients remains poor.^2^ Lenvatinib, a kind of multi-kinase inhibitors targeting the fibroblast growth factor receptor (FGFR), platelet-derived growth factor receptor (PDFGR), vascular endothelial growth factor receptor (VEGFR) et.al, has been approved for first-line treatment of unresectable HCC.^3^ However, some patients exhibit poor sensitivity or resistance to Lenvatinib, limiting its therapeutic efficacy and clinical application. Therefore, exploring new therapeutic targets to enhance the therapeutic sensitivity of HCC to Lenvatinib is crucial for improving patient outcomes.

Pyroptosis, a novel form of inflammatory programmed cell death, differs from apoptosis or necrosis.^4^ It is characterized by cell swelling, membrane perforation, the release of cellular contents and activation of an inflammatory response.^5^ Pyroptosis is executed by the gasdermin (GSDM) protein family, which includes six members: GSDMA, GSDMB, GSDMC, GSDMD, GSDME/DFNA5, and PJVK/DFNB59. With the exception of PJVK, all members contain two conserved domains: the N-terminal pore-forming domain and the C-terminal inhibitory domain.^6^ Upon stimulation by various external or internal factors, GSDMs are cleaved by specific caspases or granzymes, leading to the release of the N-terminal domain, which oligomerizes at the plasma membrane, forming large transmembrane pores. This process results in membrane rupture and the release of inflammatory molecules.^5,7,8^ Previous research on pyroptosis has primarily focused on GSDMD.^9–11^ Further studies have shown that each GSDM can be activated by specific proteases in response to distinct stimuli.^12–17^ For instance, Dasatinib has been shown to upregulate GSDMD and GSDME protein levels in lung cancer and neuroblastoma cells, inducing caspase-3/GSDME cleavage and pyroptosis.^18^ Moreover, in vitro and in vivo studies have revealed that GSDME enhances the sensitivity of cancer to targeted therapies, such as Trametinib, Erlotinib, and Ceritinib.^19^ In HCC, miltirone has been found to induce GSDME cleavage in HCC cells, causing pyroptosis and reducing cell viability, whereas GSDME knockout shifts miltirone-induced cell death from pyroptosis to apoptosis.^20^ Additionally, another natural compound, Neobavaisoflavone, induces reactive oxygen species generation in HCC cells, affecting Tom20 protein expression and promoting Bax recruitment to mitochondria, which activates caspase-3, cleaves GSDME, and induces pyroptosis.^21^ These findings suggest that GSDME-mediated pyroptosis is a crucial pathway for the anticancer effects of various small molecules, and targeting pyroptosis may be a promising therapeutic strategy for HCC. Although previous studies had revealed that Lenvatinib could induce GSDME-mediated pyroptosis,^22,23^ the underlying mechanisms and whether GSDME-mediated pyroptosis induced by lenvatinib can be used as an anti-tumor strategy remain unexplored.

S-palmitoylation (commonly referred to as palmitoylation) is a reversible post-translational modification of proteins catalyzed by palmitoyl transferases (Aspartate-histidine-histidine-cysteine palmitoyl S-acyltransferases, DHHC-PATs).^24,25^ This process involves the covalent attachment of a 16-carbon palmitic acid to the cysteine residues of proteins via an unstable thioester bond.^24,25^ Palmitoylation regulates protein trafficking and intracellular localization, as the hydrophobic palmitoyl group acts as a lipid anchor, increasing the lipophilicity of proteins and promoting their interaction with the plasma membrane. This modification also regulates the movement of proteins between the Golgi apparatus, endoplasmic reticulum, plasma membrane, and endosomal system.^26^ Palmitoylation ensures the correct localization of various intracellular proteins to the plasma membrane, where they perform essential functions.^27–29^ Additionally, palmitoylation participates in regulating protein stability, either promoting or inhibiting protein degradation.^30^ Previous studies have shown that GSDMD undergoes palmitoylation, with the palmitoylated GSDMD-N terminal promoting its translocation to the plasma membrane.^31,32^ Given that GSDME functions similarly at the plasma membrane, further exploration is required to determine whether its translocation involves palmitoylation and how palmitoylation regulates its activity.

In this study, we explored whether Lenvatinib can induce pyroptosis in HCC cells. Further experiments were conducted to determine whether Lenvatinib mediates pyroptosis via GSDME and to elucidate the underlying mechanisms. Additionally, we investigated whether interfering with GSDME, both in vitro and in vivo, could affect Lenvatinib-induced pyroptosis and subsequently alter the therapeutic sensitivity of HCC to Lenvatinib.

## Results

### Lenvatinib induces pyroptosis in HCC cells

**Figure 1.**
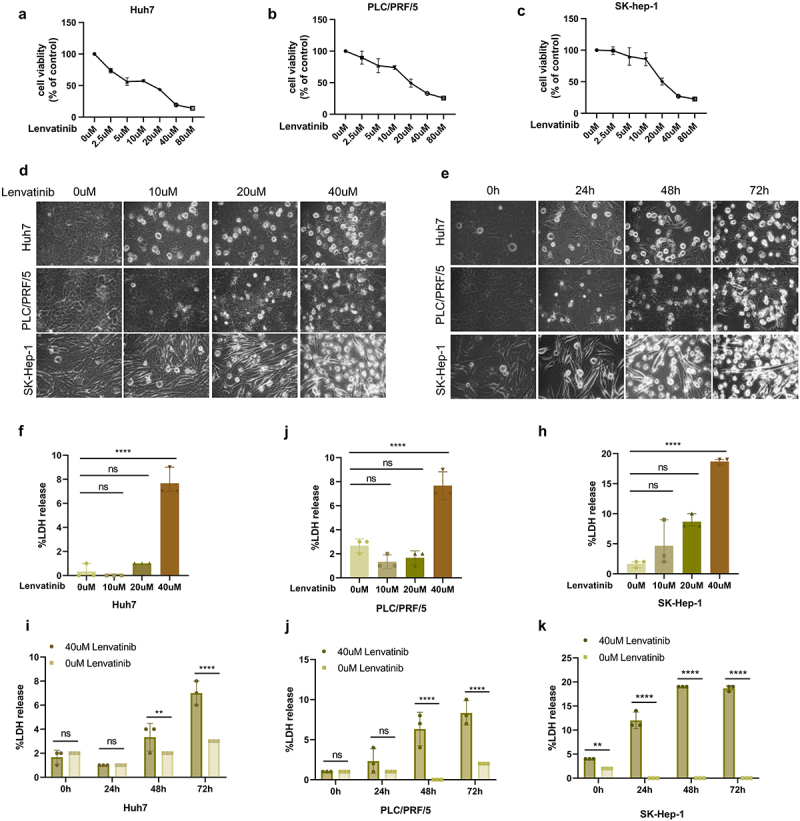
Lenvatinib induces pyroptosis in hepatocellular carcinoma (HCC) cells.

Based on the previous studies and preliminary experiments,^33^ three HCC cell lines (Huh7, PLC/PRF/5, SK-Hep-1) were treated with various concentrations of Lenvatinib (0 μM, 2.5 μM, 5 μM, 10 μM, 20 μM, 40 μM, 80 μM) for 72 hours to determine the appropriate drug concentration. Cell viability was subsequently assessed using CCK8 assays, which demonstrated a dose-dependent decrease in cell viability across all three HCC cell lines following Lenvatinib treatment. Notably, at a concentration of 40 μM, the viability of these HCC cell lines fell below 50% (Figure 1a-c). Therefore, 40 μM was chosen as the maximum concentration for subsequent in vitro experiments, with concentration gradients set at 0 μM, 10 μM, 20 μM, and 40 μM.

a-c. The cell viability of Huh7 (a)、PLC/PRF/5 (b)、SK-Hep-1 (c) were detected by CCK8 assays after treated with different concentrations of Lenvatinib for 72 h. d. The morphological changes of Huh、PLC/PRF/5、SK-Hep-1 were observed under phase contrast microscope after treated with different concentrations of Lenvatinib for 72 h. e. The morphological changes of Huh、PLC/PRF/5、SK-Hep-1 were observed under phase contrast microscope after treated with 40uM Lenvatinib for 0、24、48、72 h. f-h LDH release of Huh7 (f)、PLC/PRF/5 (g)、SK-Hep-1 (h) were detected after treated with different concentrations of Lenvatinib for 72 h. i-k. LDH release of Huh7 (i)、PLC/PRF/5 (j)、SK-Hep-1 (k) were detected after treated with 40uM Lenvatinib for 0、24、48、72 h. ns represents *P*>0.05. * represents *p* < .05, ** represents *p* < .01, *** represents *p* < .001, and **** represents *p* < .0001.

To investigate whether Lenvatinib induces pyroptosis in HCC cells, these cell lines were treated with the aforementioned concentration gradients of Lenvatinib for 72 hours and the cell morphological changes were observed under a phase contrast microscope. The results revealed significant cell swelling and the appearance of pyroptotic bubbles, both hallmark features of pyroptosis, in the Lenvatinib-treated HCC cell lines. Furthermore, the number of bubbles increased in a concentration-dependent manner (Figure 1d). Subsequently, Additionally, cells treated with 40 μM Lenvatinib were analyzed over time (0 h, 24 h, 48 h, and 72 h), and the results showed that the number of pyroptotic bubbles increased with longer treatment durations (Figure 1e).

When the GSDME-mediated pyroptosis pathway is activated, the N-terminal of GSDME forms pores at the plasma membrane, leading to the release of intracellular contents such as lactate dehydrogenase (LDH) into the extracellular space. Therefore, LDH release is commonly used as an indicator of pyroptosis. HCC cell lines treated with varying concentrations of Lenvatinib for 72 hours showed a dose-dependent increase in LDH release (Figure 1f-h). Moreover, LDH release also increased with extended treatment times (Figure 1i-k).

These findings suggest that Lenvatinib induces pyroptosis in HCC cells, with the degree of pyroptosis increasing both with higher concentrations of Lenvatinib and longer treatment durations.

### Lenvatinib induces the cleavage of GSDME in HCC

**Figure 2.**
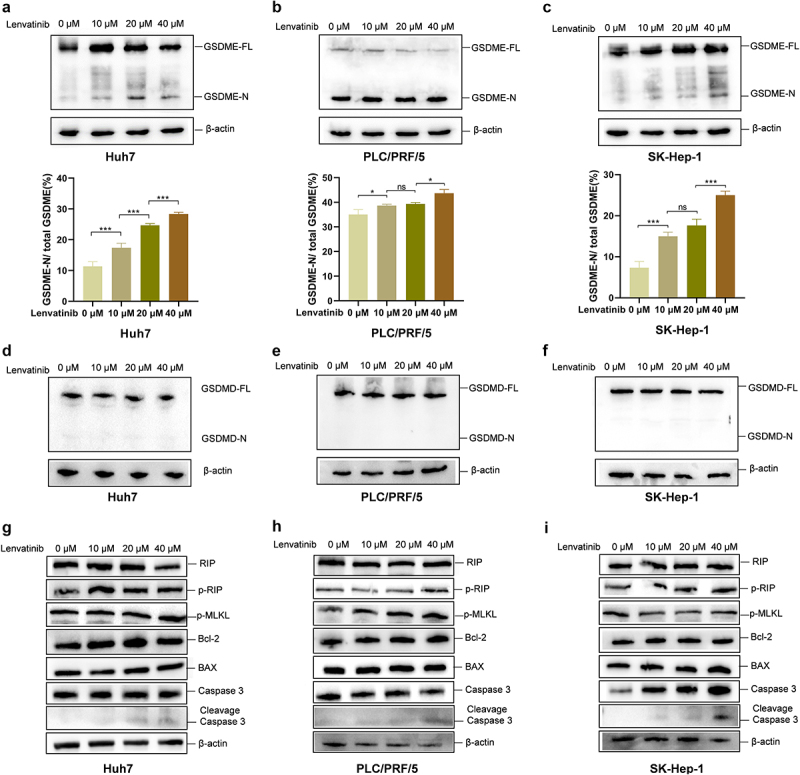
Lenvatinib induces the cleavage of GSDME in HCC.

In our preliminary experiments, we observed that Lenvatinib induces pyroptosis in HCC cell lines. Previous studies have demonstrated that other kinase inhibitors such as Dasatinib could activate GSDME-mediated pyroptosis.^18^ Since Lenvatinib is also a kinase inhibitor, we hypothesized that it might induce pyroptosis in HCC cells through the activation of GSDME. To confirm our hypothesis, Huh7, PLC/PRF/5, and SK-Hep-1 cells were treated with Lenvatinib and the expression of GSDME and its N-terminal fragment in these cells were detected by Western Blot analysis. The results revealed that Lenvatinib treatment led to a dose-dependent increase in the proportion of cleaved GSDME N-terminal fragment relative to total GSDME (Figure 2a-c), indicating that Lenvatinib promotes GSDME cleavage. Furthermore, given that both GSDMD and GSDME are widely expressed in many tissues and cells and that various stimuli could activate GSDMD to induce pyroptosis,^34^ we also investigated whether Lenvatinib could activate GSDMD-mediated pyroptosis. Western Blot analysis results showed that the N-terminal of GSDMD was not detectable following Lenvatinib treatment, indicating that GSDMD did not undergo cleavage (Figure 2d-f).

a-c. Protein expression of GSDME and its N-terminal inHuh7 (a)、PLC/PRF/5 (b)、SK-Hep-1 (c) were detected by Western Blot after treated with different concentrations of Lenvatinib for 72 h. d-f. Protein expression of GSDMD and its N-terminal in Huh7(d)、PLC/PRF/5 (e)、SK-Hep-1 (f) were detected by Western Blot after treated with different concentrations of Lenvatinib for 72 h. h-j. Protein expression of RIP, phosphorylated RIP, phosphorylated MLKL, BCL-2, BAX, caspase 3 and cleavage caspase 3 in Huh7(h)、PLC/PRF/5 (i)、SK-Hep-1 (j) were detected by Western Blot after treated with different concentrations of Lenvatinib for 72 h. ns represents *P*>0.05. * represents *p* < .05, ** represents *p* < .01, *** represents *p* < .001, and **** represents *p* < .0001.

To assess whether other forms of cell death, such as apoptosis or necroptosis, might also might also contribute, we examined the expression of classical markers associated with these pathways. Western Blot analysis showed that the levels of apoptotic proteins BAX and BCL2, as well as necroptotic markers RIPK1 and phosphorylated MLKL (p-MLKL), remained unchanged following Lenvatinib treatment in HCC cells (Figure 2g-j). In contrast, cleaved caspase-3 was upregulated in a dose-dependent manner, while total caspase-3 levels remained stable, suggesting that caspase-3 activation occurred without fully initiating a classical apoptotic cascade. These results are consistent with the non-apoptotic role of caspase-3 in cleaving GSDME and initiating pyroptosis.^35^

Collectively, these findings indicate that Lenvatinib induces pyroptosis in HCC cells primarily through caspase-3–mediated cleavage of GSDME.

### Lenvatinib induces pyroptosis in HCC cells via GSDME

**Figure 3.**
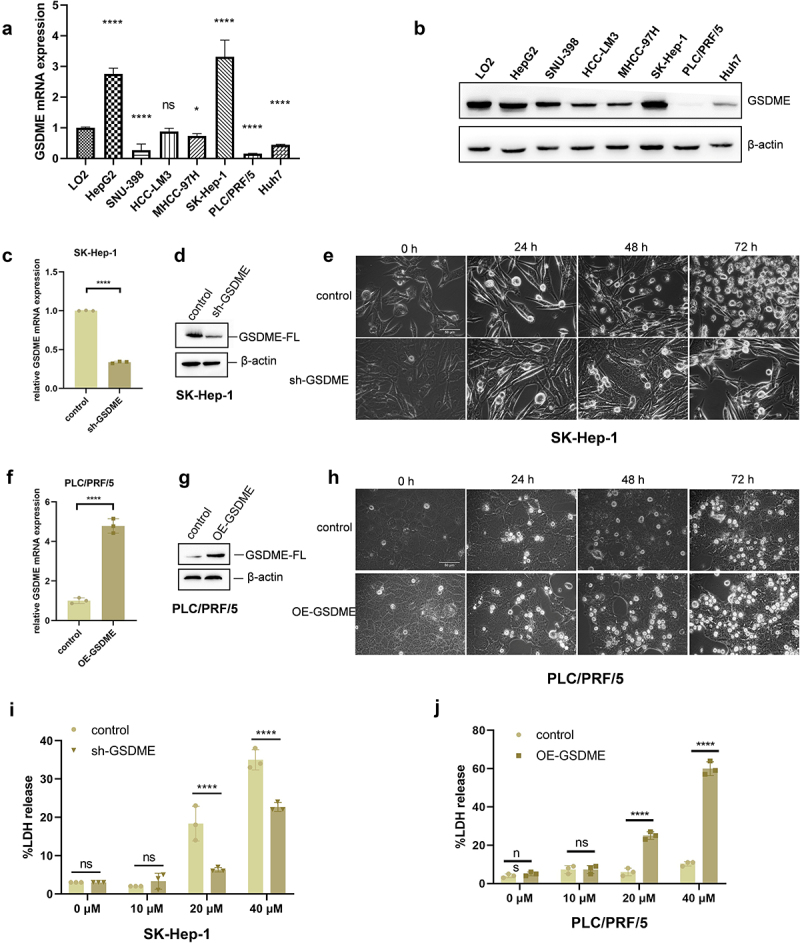
Lenvatinib induces pyroptosis in HCC cells via GSDME.

To further explore whether Lenvatinib induces pyroptosis through GSDME, we employed both loss-of-function and gain-of-function strategies to examine the role of GSDME in Lenvatinib-induced pyroptosis in vitro. First, q-RT PCR and Western Blot was conducted to assess the mRNA and protein expression levels of GSDME in normal liver cell lines and various HCC cell lines. The results showed that, except for SK-Hep-1, which had higher mRNA and protein expression levels than the normal liver cell line LO2, the mRNA and protein expression levels of GSDME were reduced in most HCC cell lines. Among these, PLC/PRF/5 cells displayed the lowest mRNA and protein expression levels of GSDME (Figure 3a-b). Based on these results, SK-Hep-1 cells were selected for GSDME downregulation, while PLC/PRF/5 cells were selected for GSDME upregulation using recombinant lentiviral technology. The efficiency of GSDME interference by the recombinant lentivirus was confirmed by q-RT PCR and Western Blot analysis (Figure 3c,d,f and g).

a. mRNA expression of GSDME in normal liver cell and various HCC cell lines were detected by qRT-PCR. b. Protein expression of GSDME in normal liver cell and various HCC cell lines were detected by Western Blot. c-d. The knockdown efficiency of GSDME in SK-Hep-1 cells was detected by qRT-PCR (c) and Western Blot (d). e. The morphological changes of SK-Hep-1 control and SK-Hep-1 sh-GSDME cells were observed under phase contrast microscope after treated with 40uM Lenvatinib for 0、24、48、72 h. f-g. The overexpression efficiency of GSDME in PLC/PRF/5 cells was detected by qRT-PCR (F) and Western Blot (g). h. The morphological changes of PLC/PRF/5 control and PLC/PRF/5 OE-GSDME cells were observed under phase contrast microscope after treated with 40uM Lenvatinib for 0、24、48、72 h. I. LDH release of SK-Hep-1 control and SK-Hep-1 sh-GSDME cells were detected after treated with different concentrations of Lenvatinib for 72 h. J. LDH release of PLC/PRF/5 control and PLC/PRF/5 OE-GSDME cells were detected after treated with different concentrations of Lenvatinib for 72 h. ns represents *P*>0.05. * represents *p* < .05, ** represents *p* < .01, *** represents *p* < .001, and **** represents *p* < .0001.

Following Lenvatinib treatment, the number of pyroptotic bubbles observed in SK-Hep-1 cells with GSDME knockdown (sh-GSDME) was lower compared to the control group (Figure 3e). Consistent with this, LDH release assays showed that the proportion of LDH release in SK-Hep-1 sh-GSDME cells was reduced relative to control cells (Figure 3i). Conversely, in PLC/PRF/5 cells overexpressing GSDME (OE-GSDME), the number of pyroptotic bubbles was significantly higher than in the control group (Figure 3h). LDH release assays also revealed an increased proportion of LDH release in the PLC/PRF/5 OE-GSDME cells compared to the control cells (Figure 3j).

These findings suggest that Lenvatinib induces pyroptosis through GSDME. GSDME knockdown inhibits Lenvatinib-induced pyroptosis, while GSDME overexpression enhances it.

### Lenvatinib enhances the plasma membrane localization and stability of GSDME by promoting its palmitoylation

**Figure 4.**
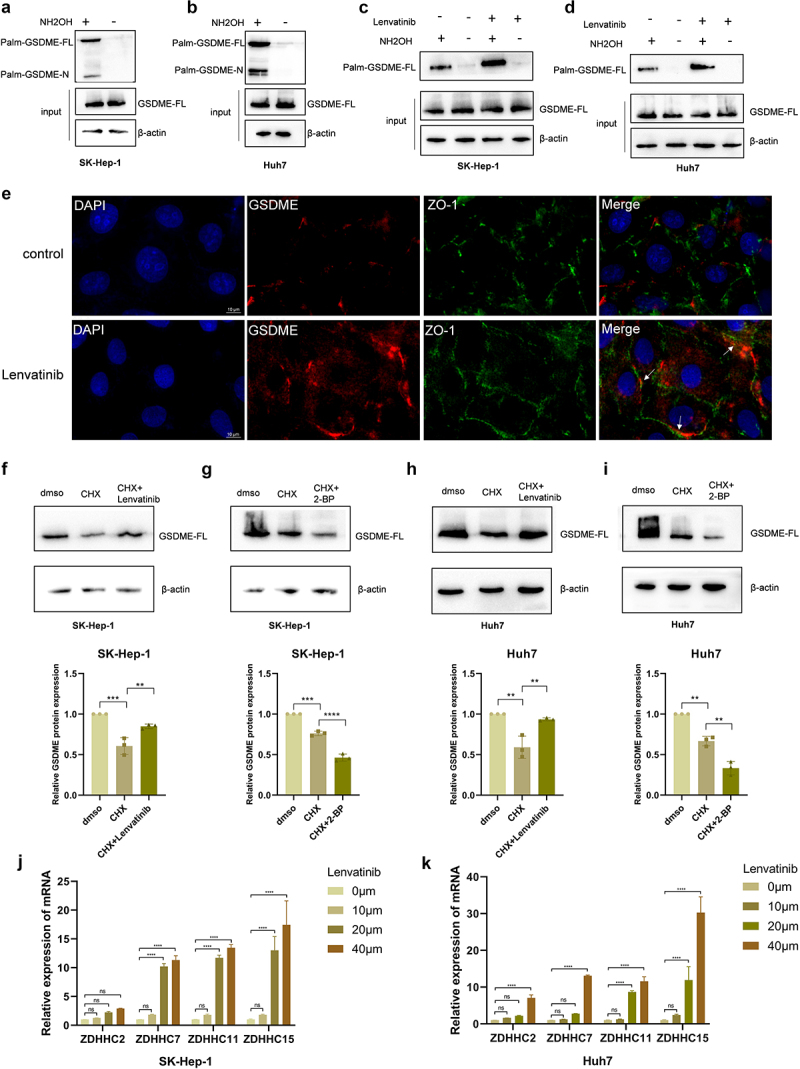
Lenvatinib enhances the plasma membrane localization and stability of GSDME by promoting its palmitoylation.

Protein trafficking is a tightly regulated process to ensure that proteins are correctly transported to destination. Palmitoylation is required for plenty of proteins to shuttle among the Golgi apparatus, plasma membrane, and endosomal system.^36^ Previous studies have found that the C-terminal of GSDME may undergo palmitoylation.^37,38^ Considering that its N-terminal need to be transported to the plasma membrane, it remains unclear whether palmitoylation is required for this transport. To address this, ABE method combined with Western Blot were performed to detect the palmitoylation of GSDME. The results confirmed that both GSDME and its N-terminal undergo palmitoylation (Figure 4a-b). Furthermore, when HCC cells were treated with Lenvatinib, the level of GSDME palmitoylation increased significantly compared to the control group (Figure 4c-d), indicating that Lenvatinib promotes GSDME palmitoylation.

a. The palmitoylation of GSDME in SK-Hep-1 cells was detected by ABE method. b. The palmitoylation of GSDME in HuH7 cells was detected by ABE method. c. The palmitoylation of GSDME in SK-Hep-1 cells was detected by ABE method after treated with 20uM Lenvatinib or not. d. The palmitoylation of GSDME in HuH7 cells was detected by ABE method after treated with 20uM Lenvatinib or not. e. The localization of GSDME was detected by immunofluorescence assays after Huh7 cells were treated with 20uM Lenvatinib or not (GSDME was marked by GSDME antibody, plasma membrane was marked by ZO-1 antibody, cell nucleus was marked by DAPI). f. SK-Hep-1 cells were treated with Lenvatinib (20 μM) for 72 h or not, and then stimulated with the addition of CHX (100 μg/ml) for 12 h. Western Blot was used to detect the protein expression of GSDME. g. SK-Hep-1 cells were treated with 2-BP (20 μM) for 72 h or not, and then stimulated with the addition of CHX (100 μg/ml) for 12 h. Western Blot was used to detect the protein expression of GSDME. h. Huh7 cells were treated with lenvatinib (20 μM) for 72 h or not, and then stimulated with the addition of CHX (100 μg/ml) for 12 h. Western Blot was used to detect the protein expression of GSDME. i. Huh7 cells were treated with 2-BP (20 μM) for 72 h or not, and then stimulated with the addition of CHX (100 μg/ml) for 12 h. Western Blot was used to detect the protein expression of GSDME. j-k. mRNA expression of ZDHHC2, ZDHHC7, ZDHHC11, ZDHHC15 in SK-Hep-1 cells (j) and Huh7 cells (k) were detected by qRT-PCR. ns represents *P*>0.05. * represents *p* < .05, ** represents *p* < .01, *** represents *p* < .001, and **** represents *p* < .0001.

To further explore the functional implications of palmitoylation on the function of GSDME, immunofluorescence experiments were performed, and the results showed that GSDME was mainly distributed in the cytoplasm in the Huh7 control group, whereas, in Lenvatinib-treated cells, GSDME accumulated more at the plasma membrane (Figure 4e). This suggested that Lenvatinib enhanced the palmitoylation of GSDME, promoting its membrane localization. In addition to influencing protein trafficking, palmitoylation also affects protein stability, promoting or inhibiting the degradation of different proteins.^37^ To investigate whether GSDME palmitoylation affects its protein stability, we treated HCC cells with Lenvatinib and then inhibited protein synthesis by using Cycloheximide (CHX), a eukaryote protein synthesis inhibitor. Compared to the Lenvatinib + CHX group, the degradation of GSDME was higher in the CHX group (Figure 4f,h), suggesting that Lenvatinib enhanced the protein stability of GSDME. Conversely, when HCC cells were treated with 2-BP, GSDME protein expression further decreased in the CHX + 2-BP group compared to the CHX group (Figure 4g,i), indicating that inhibiting GSDME palmitoylation promoted its degradation. The enzymatic process of palmitoylation is carried out by DHHC-domain-containing proteins, and it was reported that ZDHHC-2/6/7/11/15 were potential enzymes to palmitoylate GSDME.^39^ To determine whether Lenvatinib regulates GSDME palmitoylation via the aforementioned ZDHHC enzymes, we conducted qRT-PCR analysis following Lenvatinib treatment. The results demonstrated a dose-dependent upregulation of mRNA expression for four ZDHHC enzymes (Figure 4j,k), indicating that Lenvatinib may enhance GSDME palmitoylation by promoting the transcriptional activation of ZDHHC enzymes.

These findings demonstrate that Lenvatinib enhances GSDME localization at the plasma membrane and increases its protein stability by promoting palmitoylation.

### Interfering with GSDME palmitoylation influences Lenvatinib-induced pyroptosis

**Figure 5.**
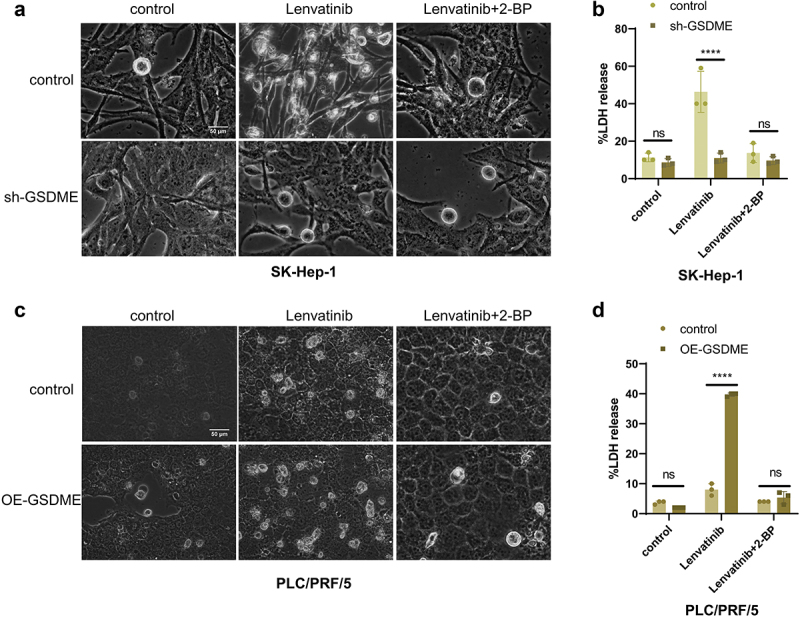
Interfering with GSDME palmitoylation influences Lenvatinib-induced pyroptosis.

To investigate the role of GSDME palmitoylation in Lenvatinib-induced pyroptosis, phase contrast microscopy revealed that in SK-Hep-1 control cells, the number of pyroptotic bubbles in the Lenvatinib-treated group was higher than in the Lenvatinib + 2-BP group. In SK-Hep-1 sh-GSDME cells, no significant difference was observed in the number of pyroptotic bubbles between the Lenvatinib-treated and Lenvatinib + 2-BP groups (Figure 5a). Similarly, in PLC/PRF/5 control and OE-GSDME cells, the number of pyroptotic bubbles in the Lenvatinib-treated group exceeded that in the Lenvatinib + 2-BP group (Figure 5c).

a. SK-Hep-1 control and SK-Hep-1 sh-GSDME cells were treated with Lenvatinib (40 μM) or Lenvatinib (40 μM) in combination with 2-BP (20 μM) for 72 h, and the morphological changes were observed by phase contrast microscopy. b. SK-Hep-1 control and SK-Hep-1 sh-GSDME cells were treated with Lenvatinib (40 μM) or Lenvatinib (40 μM) in combination with 2-BP (20 μM) for 72 h, and LDH release in each group was measured by LDH release assays. c. PLC/PRF/5 control and PLC/PRF/5 OE-GSDME cells were treated with Lenvatinib (40 μM) or Lenvatinib (40 μM) in combination with 2-BP (20 μM) for 72 h, and the morphological changes were observed by phase contrast microscopy. d. PLC/PRF/5 control and PLC/PRF/5 OE-GSDME cells were treated with lenvatinib (40 μM) or Lenvatinib (40 μM) in combination with 2-BP (20 μM) for 72 h, and LDH release in each group was measured by LDH release assays. ns represents *P*>0.05. * represents *p* < .05, ** represents *p* < .01, *** represents *p* < .001, and **** represents *p* < .0001.

LDH release assays further supported these findings. In SK-Hep-1 control cells, Lenvatinib-treated cells exhibited a significantly higher proportion of LDH release compared to SK-Hep-1 sh-GSDME cells. However, after addition of 2-BP, there was no significant difference in LDH release between the two groups (Figure 5b). LDH release in the PLC/PRF/5 OE-GSDME cells was significantly greater than in the PLC/PRF/5 control cells following Lenvatinib treatment. Nonetheless, after the addition of 2-BP, the difference in LDH release between the two groups was no longer significant (Figure 5d).

These results suggest that disrupting GSDME palmitoylation with 2-BP effectively blocks Lenvatinib-induced pyroptosis in HCC cells.

### Interfering with GSDME changes the sensitivity of HCC to Lenvatinib treatment in vivo

**Figure 6.**
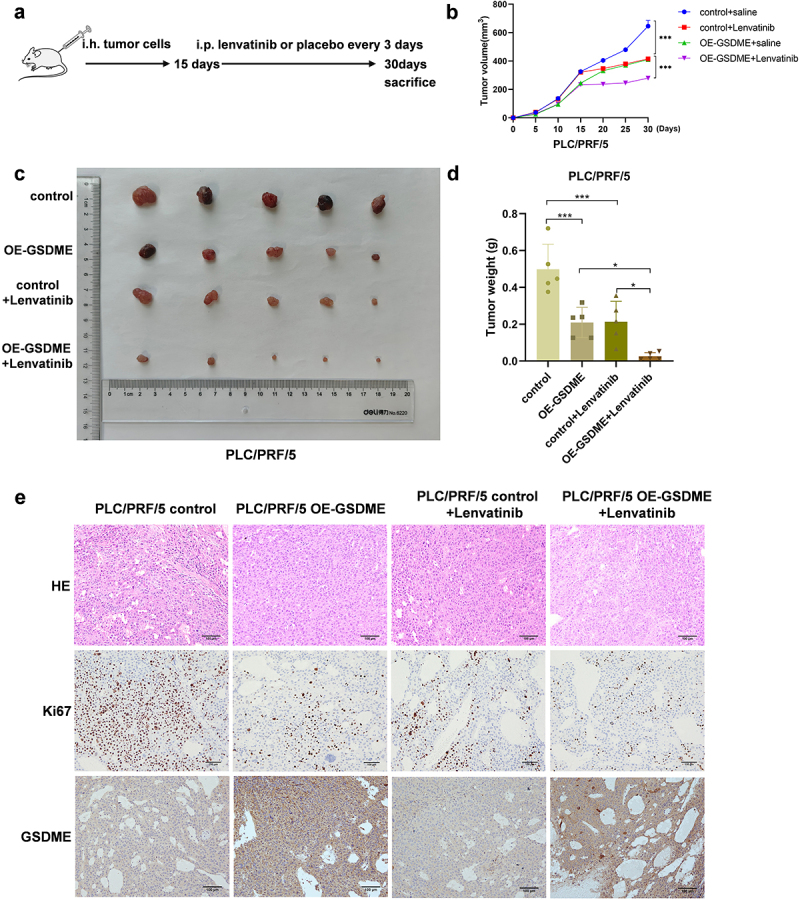
Overexpression of GSDME enhances the sensitivity of HCC to Lenvatinib treatment in vivo.

**Figure 7.**
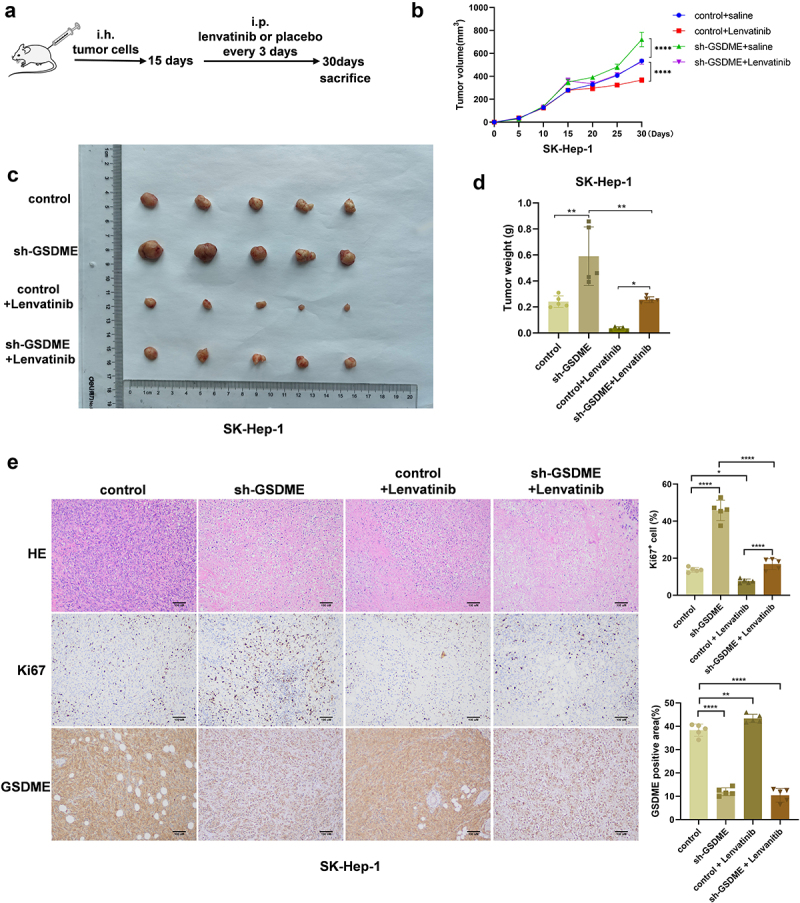
Knockdown of GSDME impairs the sensitivity of HCC to Lenvatinib treatment in vivo.

To evaluate the therapeutic potential of targeting GSDME in HCC, we constructed the mice subcutaneous xenograft model using HCC cell lines with stable GSDME overexpression (PLC/PRF/5 OE-GSDME) and their corresponding control (PLC/PRF/5 control), as well as HCC cell lines with stable GSDME downregulation (SK-Hep-1 sh-GSDME) and their control (SK-Hep-1 control). After approximately 15 days after the xenograft model was established, the mice were divided into different treatment groups (Figures 6a and 7a). Tumor size was recorded every 5 days during the experiment. At the end of the experiment, all mice were sacrificed, and tumor tissues were collected. In the PLC/PRF/5 xenograft model, tumor growth was fastest in the PLC/PRF/5 control + 0.9% saline group, which also had the largest tumor weight. Conversely, tumor growth was slowest in the PLC/PRF/5 OE-GSDME + Lenvatinib group, which had the smallest tumor weight (Figure 6b-d). In the SK-Hep-1 xenograft model, tumor growth was fastest in the SK-Hep-1 sh-GSDME +0.9% saline group, which had the largest tumor weight, whereas tumor growth was the slowest in the SK-Hep-1 control + Lenvatinib group, which had the smallest tumor weight (Figure 7b-d). IHC staining experiments confirmed the successful upregulation of GSDME expression in the PLC/PRF/5 OE-GSDME group and the downregulation of GSDME in the SK-Hep-1 sh-GSDME group. Notably, interfering with GSDME expression and Lenvatinib treatment did not significantly alter the overall histopathological characteristics of the subcutaneous tumors. In the PLC/PRF/5 xenograft model, Ki67 staining demonstrated that Lenvatinib treatment was most effective in the PLC/PRF/5 OE-GSDME group, where it significantly inhibited tumor proliferation (Figure 6e). In contrast, in the SK-Hep-1 xenograft model treated with Lenvatinib, tumor proliferation was more pronounced in the SK-Hep-1 sh-GSDME group than in the SK-Hep-1 control group (Figure 7e).

a. Experimental design of the subcutaneous HCC xenograft mice model construction and drug administration. b. Growth curves of subcutaneous tumors in each experimental group. c. Gross view of subcutaneous tumors of each experimental group at the end of the experiment. d. Subcutaneous tumor weights of mice in each experimental group. e. HE, Ki67, and GSDME staining analysis of subcutaneous tumors of mice in each experimental group (scale bar = 100 μm). ns represents *P*>0.05. * represents *p* < .05, ** represents *p* < .01, *** represents *p* < .001, and **** represents *p* < .0001.

a. Experimental design of the subcutaneous HCC xenograft mice model construction and drug administration. b. Growth curves of subcutaneous tumors in each experimental group. c. Gross view of subcutaneous tumors of each experimental group at the end of the experiment. d. Subcutaneous tumor weights of mice in each experimental group. e. HE, Ki67, and GSDME staining analysis of subcutaneous tumors of mice in each experimental group (scale bar = 100 μm). ns represents *P*>0.05. * represents *P* < 0.05, ** represents *P* < 0.01, *** represents *P* < 0.001, and **** represents *P* < 0.0001.

In conclusion, modulating GSDME expression alters the sensitivity of HCC to Lenvatinib treatment. Overexpression of GSDME enhances the sensitivity of HCC to Lenvatinib, suggesting that GSDME may serve as a promising therapeutic target for HCC.

## Discussion

In recent years, the role of pyroptosis in tumors has garnered widespread attention.^40–42^ Inducing pyroptosis in tumor cells has emerged as one of the strategies for cancer treatment.^42^ Pyroptosis is a distinct form of programmed cell death that differs fundamentally from apoptosis and necroptosis in both its morphological features and molecular mechanisms.^43^ Unlike apoptosis, which is characterized by cell shrinkage, chromatin condensation, and membrane blebbing without loss of membrane integrity, pyroptosis involves rapid plasma membrane rupture, cell swelling, and the release of intracellular contents, often accompanied by a strong inflammatory response.^43^ Necroptosis, on the other hand, is a caspase-independent pathway that relies on the activation of RIPK and MLKL to induce membrane permeabilization and lytic cell death, but without the direct involvement of gasdermins.^43^ As a member of the GSDM family, GSDME plays a significant role in inducing pyroptosis. Upon cleavage by activated caspase-3, the N-terminal fragment translocates to the plasma membrane, where it forms pores that mediate pyroptotic cell death. Previous studies have shown that GSDME is involved in chemotherapy-induced pyroptosis.^44,45^ Additionally, studies have reported that GSDME-mediated pyroptosis is closely related to the improved sensitivity to a few small-molecule multi-kinase inhibitors such as Erlotinib. Lenvatinib, a first-line treatment for advanced hepatocellular carcinoma (HCC), is generally considered to induce apoptosis as the primary mechanism of cell death.^46,47^ However, whether Lenvatinib-induced pyroptosis exerts anti-tumor effects is worthy to explore. Therefore, we aimed to explore whether Lenvatinib could induce pyroptosis and to investigate the potential role and significance of pyroptosis in the antitumor activity of Lenvatinib. In this study, we expanded the traditional viewpoint and proposed that GSDME-dependent pyroptosis is involved in Lenvatinib treatment of HCC, supported by the following evidence. First, our results showed that Lenvatinib-treated HCC cells exhibited key features of pyroptosis, including cell swelling, the formation of pyroptotic bubbles, and increased lactate dehydrogenase (LDH) release. Moreover, we found that the extent of pyroptosis in HCC cells correlated with both the concentration and duration of Lenvatinib treatment. Considering that other forms of cell death, such as necrosis, can also lead to pore formation and plasma membrane permeabilization,^48^ we further performed Western Blot analysis to clarify the specific death pathway responsible for membrane rupture. The results revealed an increase in cleaved caspase-3 expression following Lenvatinib treatment, which is consistent with its known role in mediating GSDME cleavage and pyroptotic pore formation.^31^ Notably, the expression levels of other apoptosis-related markers, including BAX and BCL2,^49^ remained unchanged, indicating that mitochondrial apoptotic signaling was not significantly activated. Moreover, necroptosis markers RIP and p-MLKL^50^ were also remained unchanged, suggesting that necroptosis was not involved in this context. Second, we demonstrated that the cleavage of GSDME in HCC cells increased in a concentration-dependent manner following Lenvatinib treatment. Besides, we sought to investigate whether Lenvatinib could induce the cleavage of GSDMD as well. Western Blot revealed that the cleavage of GSDMD did not increase with the concentration of Lenvatinib treatment. These results indicated that Lenvatinib induced the cleavage of GSDME but not GSDMD. To further confirm the role of GSDME in Lenvatinib-induced pyroptosis, we employed GSDME loss-of-function and gain-of-function strategies. Inhibition of GSDME reduced Lenvatinib-induced pyroptosis, while overexpression of GSDME enhanced it. Therefore, we concluded that as a key executor of pyroptosis, GSDME provides an essential molecular foundation for the activation of pyroptosis in Lenvatinib treated HCC cells.

As a kind of post-translational modifications, palmitoylation regulates protein trafficking between the Golgi apparatus, endoplasmic reticulum, plasma membrane, and endosomal system by altering protein hydrophobicity. Previous studies have shown that palmitoylation is required for the correct localization of various membrane proteins, such as CD36,^51^ GLUT1,^47^ and STAT3,^52^ to the plasma membrane. In 2020, a study blocked chemotherapy-induced GSDME-dependent pyroptosis by adding the palmitoylation inhibitor 2-BP to cells, indirectly suggesting that GSDME might undergo palmitoylation. They subsequently found that the palmitoylation inhibitor and the C-terminal palmitoylation site mutant increased the interaction between the C-terminal and N-terminal of GSDME, suggesting that disrupting C-terminal palmitoylation might hinder the dissociation with the N-terminal.^37^ Subsequent research confirmed the palmitoylation of the GSDME C-terminal.^38^ Given that the cleaved N-terminal of GSDME is the executor to form pores on the plasma membrane and this process involves the trafficking from the cytoplasm to the plasma membrane, we further investigated whether N-terminal undergoes palmitoylation during this trafficking process and explored the impact of Lenvatinib on GSDME palmitoylation. Through ABE experiments, we found that GSDME and its N-terminal in HCC cells were indeed palmitoylated, with the degree of palmitoylation increasing significantly after Lenvatinib treatment compared to the control group. This suggests that Lenvatinib promotes GSDME palmitoylation, facilitating its trafficking to the plasma membrane to execute its function. In addition to affecting protein trafficking and localization, palmitoylation is also involved in regulating protein stability and degradation. Many proteins such as Fas^53^ and Oct4^54^ are protected from lysosomal degradation by palmitoylation. The absence or inhibition of palmitoylation typically leads to increased lysosomal transport and subsequent protein degradation. However, for AEG-1,^55^ palmitoylation enhances its binding to the E3 ubiquitin ligase FBXW7, leading to ubiquitination and promoting protein degradation. Thus, palmitoylation have dual roles in regulating protein stability. In our study, we observed that GSDME degradation was reduced in the cycloheximide (CHX) combined with Lenvatinib group compared to the CHX-only group. However, inhibiting GSDME palmitoylation with 2-BP accelerated its degradation. This indicated that Lenvatinib promoted GSDME palmitoylation, reducing its degradation and maintaining GSDME protein stability. Furthermore, inhibiting GSDME palmitoylation reversed the enhanced pyroptosis induced by Lenvatinib in GSDME-overexpressing HCC cells, demonstrating that disrupting GSDME palmitoylation impairs Lenvatinib-induced pyroptosis in HCC cells. Furthermore, our qRT-PCR results suggest that Lenvatinib may promote GSDME palmitoylation by upregulating the expression of ZDHHC-family palmitoyl acyltransferases, as the mRNA levels of several ZDHHC enzymes increased in a dose-dependent manner following Lenvatinib treatment. However, alternative or complementary mechanisms may also be involved. Increasing evidence indicates that crosstalk between different types of post-translational modifications can influence palmitoylation efficiency. For example, phosphorylation of STAT3 has been shown to precede and facilitate its palmitoylation.^52^ It remains to be elucidated whether Lenvatinib facilitates GSDME palmitoylation by enhancing its phosphorylation or inducing conformational changes that expose key cysteine residues, thereby promoting its recognition by palmitoyl acyltransferases. Moreover, the potential involvement of depalmitoylases, such as APT1, APT2, or ABHD17 family members, should also be considered.

To explore the value of targeting GSDME for improving the sensitivity of HCC to Lenvatinib treatment, we constructed a mouse subcutaneous xenograft model using PLC/PRF/5 cells that stably overexpress GSDME and SK-Hep-1cells with GSDME knockdown. These mice were further divided into different subgroups based on whether they received Lenvatinib treatment. By analyzing the tumor growth curves, tumor volume, and tumor weight in each group, we found that for mice model with placebo treatment, overexpression of GSDME retarded the tumor growth and knockdown of GSDME promoted the tumor growth. For mice model with Lenvatinib treatment, mice with GSDME overexpression exhibited slower tumor growth, suggesting that overexpression GSDME enhancing the therapeutic sensitivity of HCC to Lenvatinib. Histological evaluation revealed that neither GSDME interference nor Lenvatinib treatment significantly affected the overall histopathological characteristics of the tumors. Ki67 staining results suggested that the Lenvatinib treatment was most effective in the GSDME overexpression group, significantly inhibiting the proliferation of tumor. Therefore, overexpression of GSDME could significantly increase the sensitivity of HCC to Lenvatinib treatment, while knockdown of GSDME impeded the therapeutic sensitivity.

However, there are several limitations in our study which require further exploration and improvement. Firstly, although we determined the IC₅₀ values of Lenvatinib in three HCC cell lines using cytotoxicity assays, the concentration range employed in our in vitro experiments still far exceeds the clinically reported trough plasma level (42.68 ng/mL) in HCC patients.^56^ Moreover, in vitro systems do not reflect the complex pharmacokinetic and metabolic environment present in vivo. Therefore, the drug concentrations used here, are while commonly employed in mechanistic studies to ensure sufficient activation of specific signaling pathways, which may have limited translational relevance to actual in vivo dosing. While our dosing strategy was guided by IC₅₀ values and we observed clear dose-dependent effects, we acknowledge that high concentrations may also induce nonspecific cytotoxicity, which represents a potential limitation of in vitro research. Future studies incorporating pharmacokinetic modeling and dose optimization in animal models will be important to bridge this gap. Another limitation of this study is that although we demonstrated that Lenvatinib promotes the palmitoylation of GSDME, we did not further identify the specific palmitoylation sites on the GSDME N-terminal domain. Given that palmitoylation is a site-specific lipid modification that can profoundly influence protein localization, membrane-binding capacity and function,^57^ elucidating the exact cysteine residues involved is critical for understanding how this modification regulates GSDME-mediated pyroptosis. In future studies, site-directed mutagenesis combined with mass spectrometry-based palmitoyl-proteomics could be employed to pinpoint the palmitoylation sites. Such investigations will help clarify whether palmitoylation directly enhances GSDME’s pore-forming activity or modulates its subcellular localization and stability, thereby refining our understanding of how Lenvatinib modulates pyroptotic signaling through post-translational regulation of GSDME. In addition, our findings establish a link between Lenvatinib-induced GSDME palmitoylation and pyroptosis, it remains unclear whether this lipid modification exerts broader effects on downstream inflammatory signaling pathways. Palmitoylation is known to play a broad role in regulating signal transduction pathways.^57^ Upon cleavage, GSDME can interact with mitochondrial and plasma membrane components, leading to membrane rupture and the release of damage-associated molecular patterns (DAMPs), which have been shown to activate the cGAS-STING pathway and induce a potent type I interferon response and inflammation.^58^ Whether palmitoylation enhances the membrane-binding affinity or pore-forming efficiency of GSDME, thereby amplifying the activation of inflammatory signaling pathways remains to be investigated. Future studies focusing on how palmitoylation modulates the biophysical properties of GSDME and its immunological consequences will provide important insights into the broader functional implications of this modification in tumor immunobiology and treatment.

## Conclusion

Lenvatinib induces pyroptosis in HCC cells, with the extent of pyroptosis increasing in a dose- and time-dependent manner. Mechanistically, Lenvatinib promotes pyroptosis by enhancing the palmitoylation of GSDME, which facilitates its localization to the plasma membrane and increases its protein stability. Both in vitro and in vivo, interference with GSDME affects Lenvatinib-induced pyroptosis, thereby altering the therapeutic sensitivity of HCC to Lenvatinib. Thus, targeting GSDME palmitoylation emerges as a potential therapeutic strategy for HCC, as it enhances the sensitivity of HCC to Lenvatinib by promoting pyroptosis.

## Materials and methods

### Reagents and materials

Lenvatinib (HY-10981), 2-Bromohexadecanoic acid (2-BP) (HY-1117702), Cycloheximide (CHX) (HY-12320) were purchased from MedChemExpress (MCE), China. BamHI, AgeI, EcoRI, Xho I restriction endonucleases were purchased from Thermo, USA. Universal antibody diluent (WB100D) was purchased from NCM biotech, China. Matrigel (356234) was purchased from Becton, Dickinson and Company, USA. Glycine (FG149) and trypsin (ET355) were purchased from Dingguo company, China. Endo-Free Plasmid Mini Kit (D6950–02) was purchased from Omega Biotek, USA. Polyethylenimine linear (PEI) (cat# 24765) was purchased from Polysciences, USA. 0.22 μm Syringe Filter (SLGP033RS) was purchased from Millipore, USA. 5 × CE II buffer (C112–01), FastPure Gel DNA Extraction Mini Kit (DC301–01), Phanta Max Super-Fidelity DNA Polymerase (P505-d1), 250kDa Plus Prestrained Protein Marker (MP202), 180kDa Plus Prestrained Protein Marker (MP201–01) were purchased from Vazyme, China.

### Cell lines and cell culture

The cell lines used in this study, including human normal hepatocytes LO2, human hepatocellular carcinoma cell lines SK-Hep-1, Huh7, MHCC-97 H, Hep3B, HepG2, PLC/PRF/5, SNU-398, and the instrumental cell line HEK293T cells were obtained from the Institute of Liver and Gastrointestinal Diseases, Tongji Hospital, Tongji Medical College, Huazhong University of Science and Technology. All above cell lines were cultured in Dulbecco’s modified Eagle medium (DMEM) complete medium containing 10% fetal bovine serum (FBS) (Cegrogen, Germany, A0500–3010). The condition of cell culture incubator is 37 ℃, containing 5% CO2.

### Plasmids and transfection

The CDS sequence of GSDME was obtained from the NCBI website, and PCR primers were designed using Snapgene and synthesized. The PCR primers were as follows (Forward: 5′- GAAGACACCGGCGGCCACGCGTGCCACCATGTTTGCCAAAGCAACCAGGAAT-3′. Reverse: 5′- AGTTTCTGCTCCATGCGGCCGCTGAATGTTCTCTGCCTAAAGCACAGAG-3′). cDNA from HCC cells were used as the template, and the target sequences were amplified using 2× Phanta Flash Master Mix (Vazyme, Nanjing, China, P510–01), and then the PCR products were purified. GSDME shRNA sequences were screened from the sigma website and synthesized (Forward: 5′- CCGGGAGCTGTTTGTGAAACAAGATCTCGAGATCTTGTTTCACAAACAGCTCTTTTTG-3′. Reverse: 5′- AATTCAAAAAGAGCTGTTTGTGAAACAAGATCTCGAGATCTTGTTTCACAAACAGCTC-3′), then the shRNA sequences were annealed. Subsequently, the PHAGE vector and pLKO.1-puro vector were digested and purified, and the PHAGE vector was homologously recombined with the GSDME target fragment, and the pLKO.1-puro vector was ligated to the shRNA sequence. The plasmid was transformed using Fast-T1 Competent Cell and subsequently extracted. For the establishment of OE-GSDME or sh-GSDME cell line, the vector PHAGE expressing GSDME (pLKO.1 expressing sh-YTHDF1), the packaging vectors psPAX and vesicular stomatitis virus-expressing envelope vector pMD2G were co-transfected in HEK293T cells for 72 h to produce lentiviral particles. After incubating HCC cells with lentivirus supernatant for 48 h, 1 µg/ml puromycin (Cayman, USA 13,884) was used to resistance screening. Western Blot was conducted to verified the interference efficiency of the recombinant lentivirus on the target gene GSDME.

### Quantitative real‑time PCR (q‑RT PCR)

After isolating and extracting RNA from cell samples by an RNA-easy isolation reagent (Vazyme, Nanjing, China, R701–02-AA), the RNA underwent reversely transcription into cDNA using HiScript® II Q Select RT SuperMix for qPCR (Vazyme, Nanjing, China, R222–01). Real-time fluorescent quantitative PCR system (Thermo, USA) was used for q-RT PCR analysis to measure relative RNA expression by using ChamQ Universal SYBR qPCR Master Mix (Vazyme, Nanjing, China, Q711). The q-RT PCR primers were as follows: GSDME forward 5′- GATCTCTGAGCACATGCAGGTC −3′, GSDME reverse 5′- GTTGGAGTCCTTGGTGACATTCC −3′. ACTIN forward 5′- CATGTACGTTGCTATCCAGGC −3′, reverse 5′- CTCCTTAATGTCACGCACGAT −3′. ZDHHC2 forward 5′- TCTTAGGCGAGCAGCCAAGGAT −3′, ZDHHC2 reverse 5′- CAGTGATGGCAGCGATCTGGTT −3′. ZDHHC7 forward 5′- GCTCTGTCTTCAGTCCATGCTC −3′, ZDHHC7 reverse 5′- AGAAGACCCTCAAGGCACAGGA −3′. ZDHHC11 forward 5′- CCTGTGTCAGTTCTCCACTCGT −3′, ZDHHC11 reverse 5′- GCACTGTCAGTTTTCATGGGCTC −3′. ZDHHC15 forward 5′- CCTGGACCTACTGGAAGTCTATC −3′, ZDHHC15 reverse 5′- CTGCTTCTGGACCTCAGGTCTT −3′.

### Western blot

Cells samples were lysed in mix of RIPA buffer with proteinase inhibitors (Promoter, Wuhan, China) and phosphatase inhibitors (Promoter, Wuhan, China). Then cell lysates were collected and centrifuged. After removing the precipitate, the protein supernatant was collected and boiled with SDS loading buffer. Subsequently, the protein samples were electrophoresed and transferred to PVDF membranes (Millipore, Germany, IPFL00010). After blocking the membranes with NcmBlot blocking buffer (NCM biotech, China, P30500) for 20 mins, the PVDF membranes were incubated overnight at 4 °C with primary antibody. The primary antibodies included GSDME (Abcam, UK, ab215191), GSDMD (Abcam, UK, ab210070), RIP (Cell Signaling Technology, #3493), Phospho-RIP (Cell Signaling Technology, #31122), Caspase-3 (Cell Signaling Technology, #14220), BAX (Cell Signaling Technology, #2772), Bcl-2 (Cell Signaling Technology, #15071) Phospho-MLKL (Cell Signaling Technology, #18640) and β-actin (Proteintech, Wuhan, China 66,009–1-lg). After washing by TBST three times, the PVDF membranes were incubated with secondary antibodies for 1 h. The secondary antibodies included HRP goat anti-rabbit antibody (Promoter, Wuhan, China) and HRP goat anti-mouse antibody (Promoter, Wuhan, China). The images were developed with enhanced chemiluminescent reagents (NCM biotech, China, P10300) by an imaging system (Tanon 5200 Multi, China).

### Immunofuorescence assay

The cells were cultured on the coverslips in 24-well plates and after the cells reached the appropriate density, the cells were washed three times using PBS, then fixed with 4% paraformaldehyde for 20 min, and permeabilized by 0.1% Triton X-100 for 10 min. Subsequently, 10% goat serum (BOSTER, Shanghai, China) was used to block the cells for 1 h and then incubated the cells with anti-GSDME (Proteintech, Wuhan, China 13,075–1-AP) and anti-ZO-1 (Proteintech, Wuhan, China 66,452–1-lg) at 4 °C overnight. Next day, the cell slides were placed in a room temperature environment for 2 hours to rewarm, and then were washed three times using PBS. Subsequently, the cells were incubated with the Cy3-labeled Goat Anti-Rabbit IgG (H+L) antibody and Alex Fluor 488 Goat Anti-Mouse IgG(H+L) antibody (Promoter, Wuhan, China) in an environment protected from light for 2 h. Cell nucleus were stained with DAPI for 7 min and then were washed three times using PBS. Cell fluorescence images were observed using a fluorescence microscope.

### Cell viability assay

Cells were seeded in 96-well plates and treated as indicated. The cell viability was measured using a Cell Counting Kit-8 (DOJINDO, Japan, CK12) according to the protocol. Cell viability was calculated according to the following formula, Cell viability (%) = [A – B] ÷ [C – B] × 100%. A: the treated groups; B: the blank groups; C: the untreated groups.

### LDH release assay

Cells were seeded in 96-well plates and treated as indicated. The release of LDH was measured using an LDH assay kit (DOJINDO, Japan, CK12) according to the protocol. The LDH release rate of each group according to the following formula. Cellular LDH release rate (%) = [(A-C)/(B-C)] × 100% A: absorbance of the experimental group; B: absorbance of the high control; C: absorbance of the low control.

### Acyl-biotin exchange (ABE)

Cells were seeded in 10 cm cell culture dishes and treated as indicated. The palmitoylation of GSDME was detected using an CAPTUREome TM S-Palmitoylated Protein Kit (Badrilla, UK, K010–311) according to the protocol. Briefly, the sulfhydryl groups in proteins were first sequestered, followed by cleavage of the thioester bonds using hydroxylamine, then selective labeling of palmitoylated cysteines using a thiol-reactive biotinylated reagent, followed by protein pull-down using biotin-antibody-coupled agarose beads, and later analyzing the palmitoylation of the proteins in each sample through Western Blot.

### Animal experiments

All animal experimental procedures and operations in Laboratory Animal Center, Tongji Hospital, Tongji Medical College, Huazhong University of Science and Technology have been reviewed and approved by the Laboratory Animal Welfare Ethics Committee of Tongji Hospital, Tongji Medical College, Huazhong University of Science and Technology. In accordance with the three R’s and the requirements of the Ethics Committee 51 Balb/c nude mice were purchased from Vitalriver, BeiJing, China, housed in groups of 5 per cage under controlled conditions.Animals were kept in a temperature-controlled environment (22–24°C), with a 12-hour light/dark cycle. All mice were monitored for signs of illness or distress, and those showing signs were excluded. Experiments began after one week of adaptive feeding. Mice were randomly assigned to treatment or control groups using a computer-generated randomization sequence. Twelve four-week-old BALB/c male nude mice were subcutaneously injected with 5 × 10^6^ PLC/PRF/5 control cells, and 13 mice injected with PLC/PRF/5 OE-GSDME cells, and 13 mice injected with SK-Hep-1 control cells, and 13 mice injected with SK-Hep-1 sh-GSDME cells. These cells were respectively premixed with 50 µl Matrigel (Corning, 354,234) in 50 µl PBS. The tumor size was measured every 5 days. After about two weeks of modeling, the mice in each group randomly assigned to treatment or control groups using a computer-generated randomization sequence, and the treatment group was injected intraperitoneally with Lenvatinib at a dose of 10 mg/kg, while the control group was injected with the same dose of saline, which was administered every three days. When the tumors have grown to an appropriate size (about 30 days), the mice were sacrificed by cervical vertebra dislocating and then the tumors were taken out for weight measurement, volume estimation (volume = 1/2×length× width2) and Immunohistochemistry (IHC) staining. Tumor volume measurements and pathological evaluations, including IHC scoring, were conducted in a blinded manner by investigators unaware of the group allocations. IHC evaluation was performed by an independent pathologist not involved in the experimental procedures. 5 animals per group were included for statistical analysis after excluding mice that did not develop tumors.

### IHC staining

The IHC staining of GSDME in tumor tissues was performed as described previously.^59^ The anti- GSDME antibody used in IHC staining was purchased from Proteintech (13075–1-AP).

### Statistical analysis

All data were analyzed using GraphPad Prism. Data were presented as mean ± standard deviation (SD). Differences between two groups were compared using Student’s t-test. For comparisons involving more than two groups, one-way ANOVA followed by Tukey’s multiple comparisons test was used. *p* > .05 was considered not statistically significant (ns). * represents *p* < .05, ** represents *p* < .01, *** represents *p* < .001, and **** represents *p* < .0001.

## Data Availability

The data used and/or analyzed during the current study are available from the corresponding author on reasonable request.

## Abbreviations

ABE: Acyl-biotin exchange
CCK8: Cell Counting Kit-8
CHX: Cycloheximide
DHHC-PATs: Aspartate-histidine-histidine-cysteine palmitoyl S-acyltransferases
DMEM: Dulbecco’s modified Eagle medium
FGFR: Fibroblast growth factor receptor
FBS: Fetal bovine serum; GSDMD, Gasdermin D
GSDME: Gasdermin E
HCC: Hepatocellular carcinoma
LDH: Lactate dehydrogenase
OE: Over expression
PBS: Phosphate buffer saline
qRT-PCR: Quantitative real-time polymerase chain reaction
PDFGR: Platelet-derived growth factor receptor
μM: μmol/L
μl: Microliter
VEGFR: Vascular endothelial growth factor receptor
2-BP: 2-Bromohexadecanoic acid.

## Arrive guidelines statement

The animal experiment conduction in this study has adhered to ARRIVE guidelines.
